# Porcine circovirus 3: a new challenge to explore

**DOI:** 10.3389/fvets.2023.1266499

**Published:** 2024-04-24

**Authors:** Rosecleer Rodrigues da Silva, Diego Ferreira da Silva, Victor Hugo da Silva, Alessandra M. M. G. de Castro

**Affiliations:** ^1^Department of Undergraduate Studies in Veterinary Medicine, Faculdade Anclivepa, São Paulo, Brazil; ^2^Postgraduate Program in Adult Health Nursing (PROESA), Escola de Enfermagem da Universidade de São Paulo (EEUSP), São Paulo, Brazil; ^3^Graduate Program in Environmental and Experimental Pathology, Universidade Paulista (UNIP), São Paulo, Brazil

**Keywords:** emerging, porcine circovirus, swine, PCR, hosts

## Abstract

The intensification of production processes, resulting from the rise in pork production, contributes to environmental changes and increased interaction between humans, animals, and wildlife. This favorable scenario promotes the spread of potent viral species, such as PCV3, increasing the potential for the emergence of new pathogenic agents and variants. These changes in the epidemiology and manifestation of PCV3 highlight the need for enhanced understanding and control. The current literature presents challenges in the classification of PCV3, with different groups proposing diverse criteria. Establishing common terminology is crucial to facilitate comparisons between studies. While consensus among experts is valuable, new approaches must be transparent and comparable to existing literature, ensuring reproducible results and proper interpretation, and positively impacting public health. This study aims to review the literature on PCV3 infection, exploring its key aspects and highlighting unanswered questions.

## Introduction

Brazil, the fourth-largest global pork producer, has witnessed a remarkable increase in production over the past four decades, rising from 1.15 million tons in 1980 to 4.95 million tons in 2022 (1). Projections indicate a further increase to 5.1 million tons by 2023, according to the Brazilian Swine Breeders Association (ABCS) and the Brazilian Animal Protein Association (ABPA) (2, 3). This expanding context is crucial for comprehending the emergence of Porcine Circovirus 3 (PCV3) as a new challenge.

The increase in pork production has a direct impact on the intensification of production processes. Coupled with the rise in the human population, urbanization, environmental alterations, and interaction with wildlife, this trend creates a favorable environment for the spread and perpetuation of potent viral species. Consequently, this scenario increases the potential of new pathogen emergence and/or variants, leading to shifts in epidemiology and disease manifestation (4–6).

Since 1980, various significant viruses were reported in pigs’ productions systems across multiple countries, including certain species of porcine circovirus (PCV). PCVs belong to the *Circovirus* genus of the *Circoviridae* family and currently encompass four species: *Porcine circovirus* 1 (PCV1), *Porcine circovirus* 2 (PCV2), *Porcine circovirus* 3 (PCV3), and *Porcine circovirus* 4 (PCV4). Among these, PCV2 is the primary emerging virus documented so far (7–9).

*Porcine circovirus* 3 (PCV3), the focus of this study, was first identified in 2015 in sows and mummified fetuses from a pig farm in North Carolina. Since then, PCV3 has been reported in various countries (10–16). It is one of the most extensively studied PCVs, second only to PCV2, as evidenced by the high number (*n* = 624) of publications on PUBMED^1^ using the term “Porcine circovirus 3” since 2016. PCV3 presents significant challenges in its classification due to the notable genetic variability and the diverse proposals for taxonomic criteria in the literature. Therefore, it is imperative to conduct in-depth research to establish common terminology and solid classification criteria, ensuring reproducible results and promoting essential advances in the understanding and management of viral diseases, with substantial impacts on animal health and potential public health ramifications (17).

## Characterization and viral diversity

PCV3, which is a member of the *Circovirus* genus within the *Circoviridae* family, has an icosahedral morphology with approximately 17 nm in diameter and is non-enveloped. Its genome is composed of a circular single-stranded DNA. Over time, PCV3 detection in swine herds has been increasing worldwide since its initial discovery. In addition, although the PCV3’s mutation rate is lower than that of PCV2, several studies have identified the classification of PCV3 into two separate subtypes (PCV3a and PCV3b) or three genotypes (PCV3a, PCV3b, and PCV3c). The PCV3 genome consists of single circular DNA strand consisting of 2000 nucleotides (nt), with two primary genes oriented in opposite directions (ambisense expression). The genome is comprised of 50% GC and features three main Open Reading Frames (ORF) (10, 11, 18).

**Figure 1:**
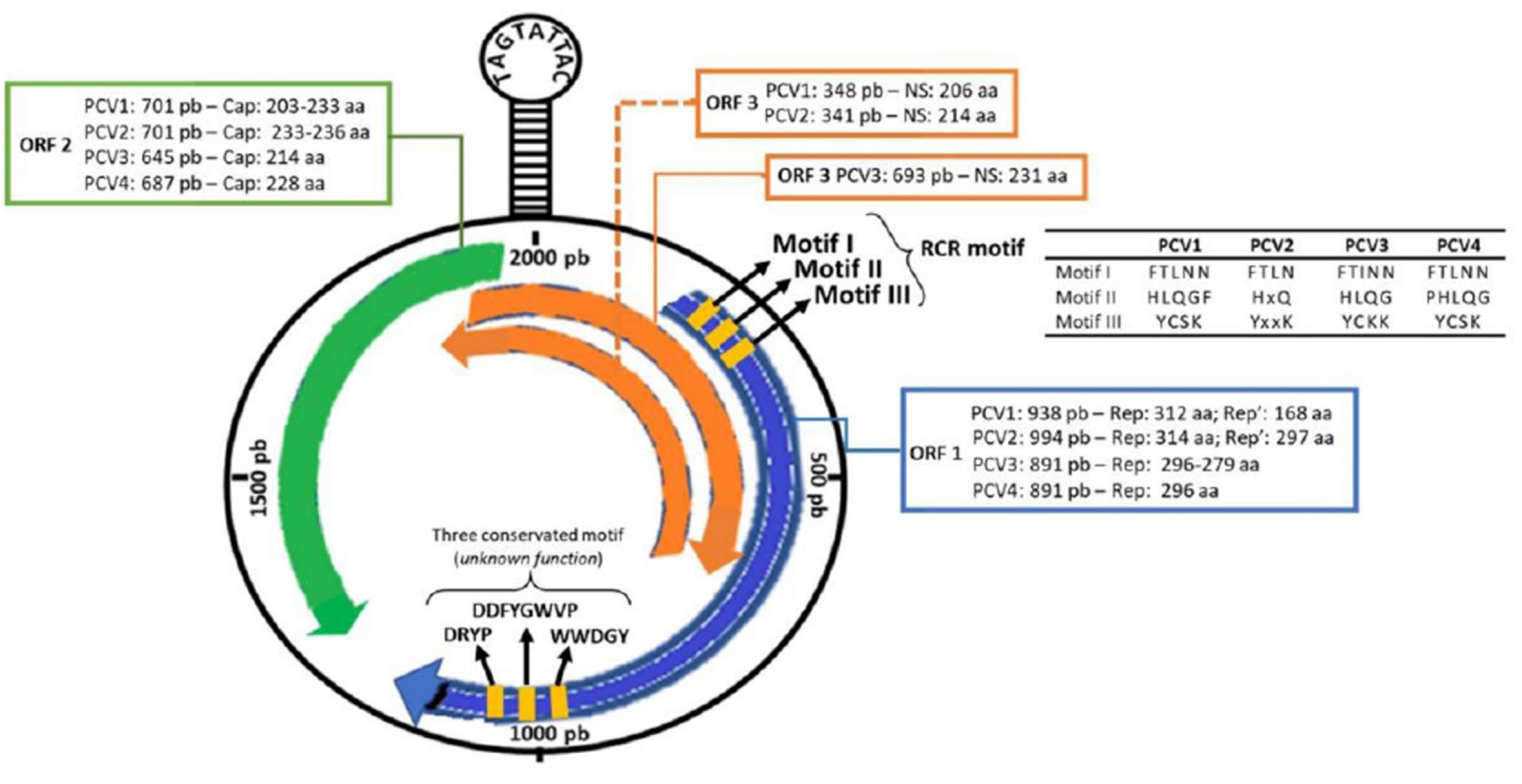
Schematic representation of the PCV3 genome. Information for comparing the open reading regions (ORF1, ORF2, ORF3) and their encoded proteins (rep, rep’ and cap) among the four porcine circovirus species (PCV1, PCV2, PCV3 and PCV4) [Updated with information from Chen et al. (19), Klaumann et al. (20), and Ouyang et al. (14)].

Like the others PCVs, the genomic arrangement of PCV3 includes two main genes, ORF1 and ORF2, which are positioned in opposite directions. ORF2 codes for the viral capsid proteins, known as the cap gene, while ORF1 codes for the replicase, or rep gene. Additionally, there is a hairpin structure (*stem-loop*) in the 5’ 235 nt intergenic region between ORF1 and ORF2 that contains a conserved sequence (TAGTATTAC). This conserved sequence serves as the origin of replication (ori) during the rolling circle replication process. A schematic representation of the PCVs genome is presented in (Figure 1) (10, 11, 21, 22).

The ORF1 gene is located on the positive strand (sense) of the circovirus genome and is known to be the most conserved region. In PCV3, ORF1 also encodes a single replicase protein consisting of 296-297 amino acids (aa). Additionally, the gene features three Rolling Circle Replication (RCR) motifs (FTINN, HLQG, and YCKK) and an initiation codon (GTC) located at the 5’ end of the rep gene (9, 11). Analysis of circovirus RCR motifs has revealed that PCV3, goose circovirus (GoCV), and pigeon circovirus (PiCV) share a degree of similarity. However, there is one mutation that has been identified in the FTLNN motif, which is present as FTINN in PCV3 (11). Although three other motifs (WWDGY, DDFGWVP, and DRYP) have been identified in PCV3, their functions remain unknown (11). Similarly, as in PCV4, the ORF1 in PCV3 encodes a replicase protein with 296 amino acids (23). ORF1 codes for the Rep and Rep’ proteins in PCV1 and PCV2. In PCV1, the Rep and Rep’ proteins consist of 312 and 168 amino acids, respectively. In PCV2, the Rep protein consists of 314 amino acids, while the Rep’ protein consists of 297 amino acids (24). Therefore, the comparison between the ORF1 of PCV3 and PCV4 reveals crucial nuances in viral research. While PCV3 features a replicase of 296-297 amino acids with three RCR motifs, PCV4 shares a configuration of 296 amino acids, resembling PCV3 (25). However, thorough investigation of RCR motifs in PCV4 is currently underway. This analysis is pivotal in unraveling the evolutionary and functional adaptations of these circoviruses, providing valuable insights for swine disease control strategies.

Located on the negative strand (antisense) of the viral DNA, ORF2 encodes only one structural protein known as Cap, which is considered the most variable and immunogenic. The Cap protein comprises 230-233 amino acids in PCV1, 233-236 amino acids in PCV2, 214 amino acids in PCV3, and 228 amino acids in PCV4. Phylogenetic analyses have shown that PCV1 and PCV2 have a 67% similarity in the Cap protein, whereas the similarity reduces to 24% between PCV1 and PCV3, and of 26 to 37% between PCV2 and PCV3 (10, 11, 26).

ORF3 displays a different sense among PCVs, as it is located on the sense strand in PCV3 and on the antisense strand in PCV1 and PCV2. In both PCV1 and PCV2, this region encodes a non-structural protein capable of inducing apoptosis, with 206 aa and 104 aa, respectively. ORF3 in PCV3 codes for a 231 aa protein, but the function and initiation codon remain unknown (10, 11). According to Ye et al. (27), the amino acid sequence of PCV3 shows homology to PCV1 and PCV2 at only 31 and 48%, respectively. No information is available on ORF3 in PCV4.

According to phylogenetic studies, the origin of PCV3 is distinct from other PCVs, and it shares a common ancestor with circoviruses found in bats. A comparative analysis of the genomics indicated that the most conserved area among PCV3, PCV2, and bat circovirus is confined to ORF1, which is responsible for encoding the rep protein. Conversely, no significant alignment was detected in ORF2, which encodes the cap protein. Despite presenting conserved segments with other circoviruses, the PCV3 ORF1 is genetically distant and has accumulated several mutations over time, indicating that the divergence between virus species occurred approximately 50 years ago (28).

It must be highlighted that PCV3 can be divided into two (PCV3a and PCV3b) (13) or three (PCV3a, PCV3b, and PCV3c) (9, 14) genotypes based on the mutation of two amino acids (A24V and R27K) found in the cap protein (11, 29). Several studies have also pointed toward the subdivision of PCV3a (13, 29–31). According to Li et al. (13), PCV3a can be divided into two stable subclades (PCV3a-1 and PCV3a-2) and an intermediate clade (PCV3a-3), which supports the subdivision proposed by Zheng et al. (29). However, a more recent study by Chen et al. (30) described, also, the division of PCV3b into two subclades (PCV3b-1 and PCV3b-2), in addition to the subclades of PCV3a (PCV3a-1, PCV3a-2, PCV3-a-3) (32). conducted a PCV3 mapping based on a viral coding gene that resulted in three genotypes and several subtypes (genotype 1, genotype 2 with subtypes a and b, genotype 3 with subtypes a–h). These findings highlight the genetic diversity of PCV3 and its tendency to increase within the global swine population, while also underscoring the challenge in establishing the pathogenesis caused by PCV3 infection (13, 33). The nomenclatures utilized thus far for PCV3 are succinctly outlined in Table 1.

**Table 1:** Taxonomic classification and distinctive features of PCV3 subgroups in samples collected Internationally 2023. (Source: Author).

**Identification**	**Country**	**Year**	**Authors**
PCV3a, PCV3b and PCV3c	China	2015 and 2017	Qi et al. (15)
		2021	Cui et al. (25)
PCV3a and PCV3b	Brazil	2006- 2007	Saraiva et al. (35)
PCV3 a1, a2, b1 and b2.	Colombia	2018	Vargas-Bermudez et al. (36)
PCV3a-1, a-2, a-3, b-1 and 3b-2	China	1996 and 2019	Chen et al. (30)
PCV3a	USA	2019	Franzo et al. (37)
PCV3-1, 3-2a and 2b, 3-3a to 3h.	Korea	2017-2018	Chung et al. (38)

Like the extensively studied PCV2, which has undergone various subclassifications (clades, genogroups and genotypes) since its discovery, PCV3 follows a comparable pattern of classification. In summary, validated criteria for defining a PCV3 genotype include a maximum genetic distance of 3% at the complete genome and 6% at ORF2 levels, as well as a bootstrap support or posterior probability on the phylogenetic tree greater than 90% (10).

Other studies (13, 15, 34) classified PCV3 into three distinct genotypes, i.e., PCV3a, 3b, and 3c. However, when aligning sequences from these genotypes, a remarkable genetic similarity to previously classified as PCV3a emerged. The maximum genetic distance observed among reported ORF2 sequences was 0.0388 (3.8%), below the recommended threshold for defining a new genotype. Hence, following established criteria, all these sequences would be categorized within the PCV3a classification. Currently, efforts have been made to standardize the classification of the PCV3 virus at the subspecies level. A standard classification will be essential for effective disease control, offering substantial benefits to animal and public health initiatives.

## Epidemiology

PCV3, which was initially identified in the United States in 2016. However, through retrospective studies, it has been detected in clinical samples from the years 60, suggesting that virus was circulating on pigs earlier their first report (39, 40). PCV3 has been identified in pig herds in several countries across the globe, including Asia (China, Korea, Japan, and Thailand), Europe (Denmark, Spain, Hungary, Ireland, Italy, Portugal, the United Kingdom, Russia, and Sweden), South America (Argentina, Brazil, Chile, and Colombia), and North America (Canada and Mexico). This widespread distribution of the virus highlights its global presence (11, 18, 41–47).

Several countries have conducted studies to determine the prevalence of viral infections using the polymerase chain reaction (PCR) technique. In Brazil, a study analyzed tissue samples from swine herds in nine states (Mato Grosso, Mato Grosso do Sul, Goiás, Minas Gerais, São Paulo, Espírito Santo, Santa Catarina, Rio Grande do Sul, and Paraná), and found that 47.8% of the samples were positive for PCV3 (35).

A study conducted in Thailand analyzed tissue and serum samples from 26 farms between 2006 and 2017 and found a 36.7% positive rate for PCV3 (48). In other countries such as Ireland, the United States, Poland, Denmark, and Sweden, positive rates ranged from 16.61 to 56.41% (11, 27, 48–50). Spain showed rates of 11.47 and 14.89% (20, 49).

**Figure 2:**
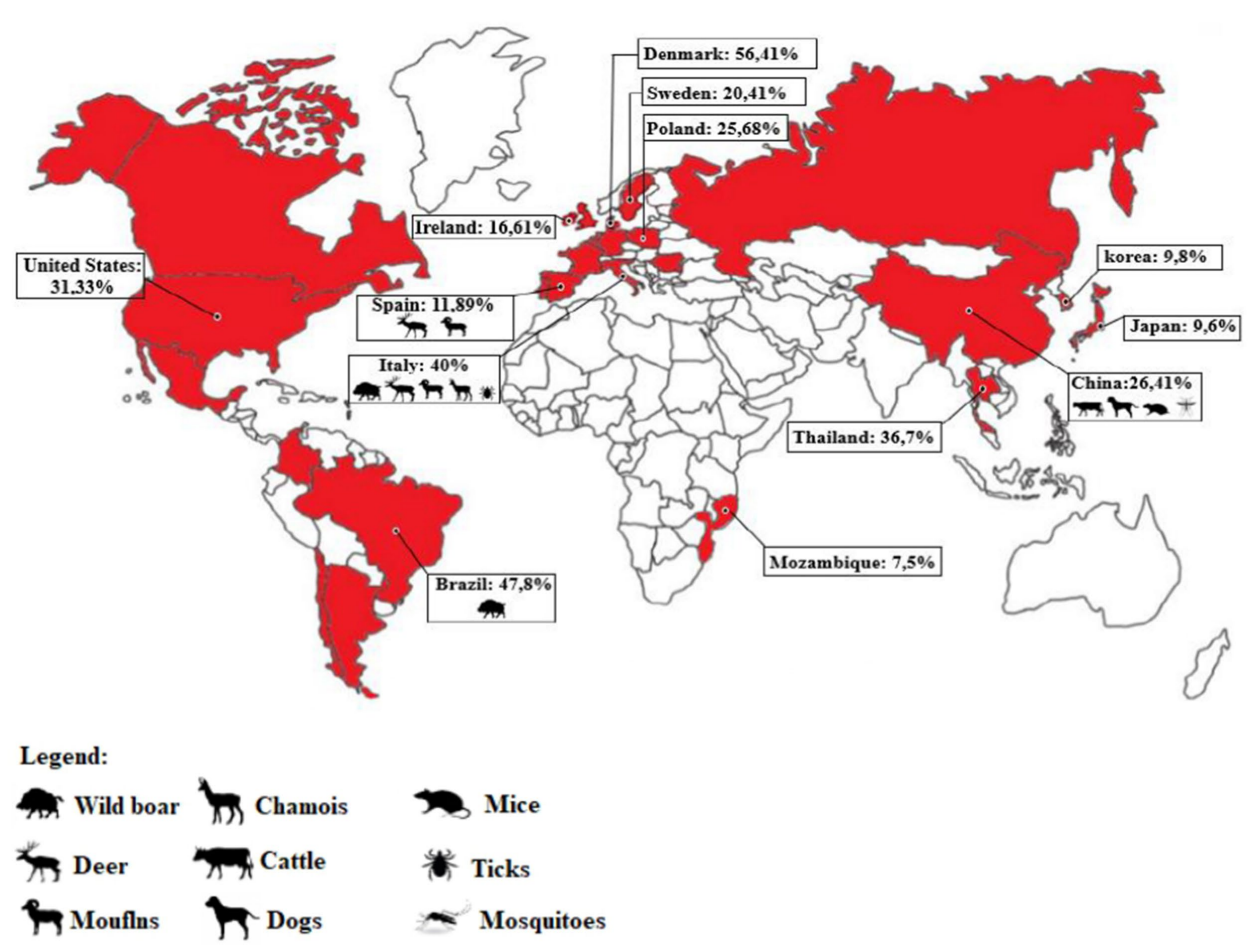
Map representation indicating the countries where PCV3 infection has been detected (highlighted in red). The percentage of positive samples in pigs and other animals’ species is also displayed for each country, based on the available literature. In cases where multiple studies were conducted in a country (such as China, Spain, and Italy), the percentage was calculated based on the total number of samples tested across all studies, to obtain an average for the country (Source: author).

According to studies by Franzo et al. (49), Italy recorded PCV3 positive rates of 39.56 and 50%, respectively. In China, there have been several studies conducted on the circulation of PCV3, with the virus being detected in more than 24 Chinese provinces, including the identification of its genotypes (PCV3a-1, PCV3a-2, PCV3a-3, PCV3b-1, and PCV3b-2) (30). Positive rates in China have shown significant fluctuations (ranging from 10 to 60%), with some farms even recording a 100% rate (13, 51–57). Anahory et al. (58) analyzed 172 archived DNA samples consisting of spleen, tonsils, liver, and ganglia collected from 91 pigs in nine of the ten provinces of Mozambique between 2011 and 2019. Their analysis provided the first evidence of PCV3 presence in Africa, with a total of 7 (7.5%) positive animals (Figure 2).

The origin of PCV3 is distinct from other PCVs, and it is believed to have evolved from the bat circovirus before adapting to pigs and other animals, allowing for cross-species transmission (10, 59). In addition to domestic pigs, PCV3 has been found in other mammals, both wild and domestic, including dogs, cattle, mice, chamois (*Rupicara rupicara*), deer (*Cervus elaphus* and *Capreolus capreolus*), and wild boar (*Sus scrofa*), as well as arthropods such as ticks (49, 60–62). In Italy, PCV3 has been detected in chamois, deer, mouflons (*Ovis musimon*), and wild boars (49).

**Figure 3:**
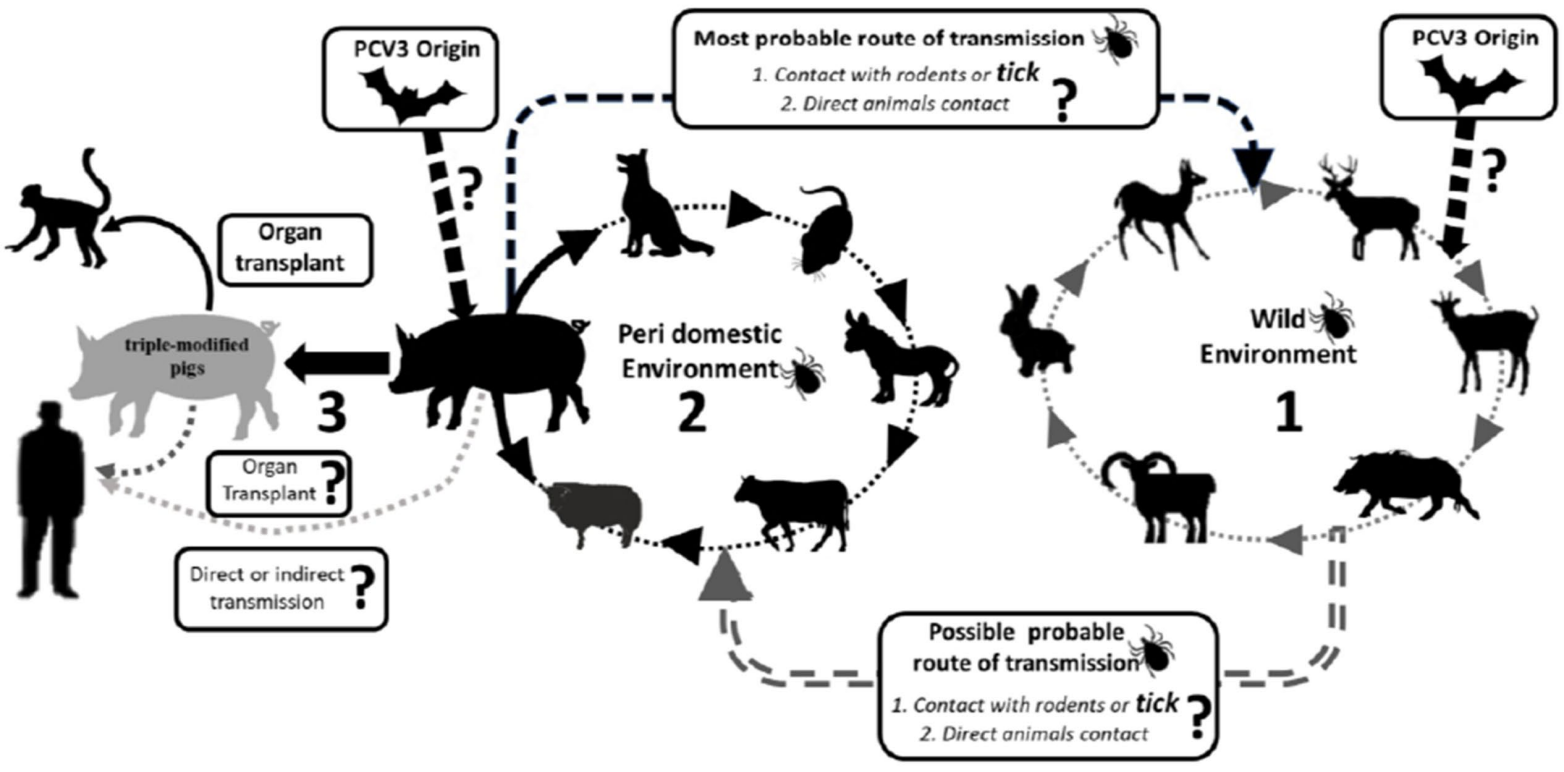
Schematic representation of potential occurrence PCV3 transmission route across different species is a potential occurrence based on the available literature. Continuous arrows indicate confirmed transmission. Dashed from commercial herds to triple-modified pigs’ herds for organ transplant.

Samples from dogs, cattle, and mice in China have also tested positive for PCV3, in addition to pigs (62, 63). In Spain, PCV3 has been detected in deer (*Cervus elaphus* and *Dama dama*) and mouflons (*Ovis aries*) (64). PCV3 has also been found in invertebrates, including ticks (*Ixodus ricinus*) in Italy and mosquitoes (*Aedes vexans*, *Anopheles sinensis*, *Culex tritaeniorhynchus*, and *Culex pipiens pallens*) in China (65, 66). Records of PCV3 presence in wild boars are reported in Brazil (67). A study conducted by Franzo et al. (49) in the Colli Euganei Regional Park of northern Italy collected 187 serum samples from wild boars and showed a high prevalence of PCV3, around 30%. Although almost all the animals were in good health, this study highlights the potential role of wild boars as a reservoir for PCV3, endangering pig farming (49). Due to the significant mutation rate of PCV3 and its discovery in various animal species, there is a concern about the likelihood of human infection. In a recent case, PCV3 was found in a herd of triple-modified pigs, which were raised for xenotransplantation. A PCV3-positve heart was transplanted into a baboon recipient and the virus was detected in all organs of the baboon recipient. The higher viral load, observed in animals with longer transplant survival times, suggested active virus replication (68) (Figure 3).

PCV3 infection can affect both, healthy and sick pig (15, 35, 52) found a strong correlation between PCV3 infection and respiratory and digestive diseases, with 26.6% of pigs with respiratory diseases and 10.4% with digestive diseases testing positive for the virus (54) also showed that PCV3 rates were significantly higher in pigs with severe respiratory disease (63.75%) or diarrhea (17.14%) compared to those with mild respiratory disease (13.14%), no diarrhea (2.86%), or no symptoms (1.85%). Furthermore, healthy sows (21.9%) and pigs from slaughterhouses (19.14%) also tested positive for PCV3 (52, 57). In Brazil, the positive rates of PCV3 were higher in healthy pigs (29.8%) compared to diseased pigs (17.9%) (35). The virus can infect pigs of all ages, including animals aged 1 day to 24 weeks, gilts, and multiparous sows (45). Therefore, the available evidence suggests that PCV3 infection is not related to the health status, sex, or age of the animal, but rather to the presence of environmental and animal condition that favor viral infection (14).

The PCV3 DNA has been detected in various tissues and fluids, including the brain, kidney, heart, spleen, serum, oral and nasal fluids, feces, colostrum, and semen of both healthy and sick animals (Table 2). Additionally, PCV3-specific antigens have been identified in the skin, lung, heart, kidney, lymph nodes, spleen, liver, and small intestine of infected piglets, both symptomatic and asymptomatic (73, 74), PCV3 DNA was detected in serum samples from healthy boars imported from the United States and Western European countries, indicating the potential for global transmission (73, 75). Vertical transmission is also a concern since the virus is widely distributed in various tissues (14).

**Table 2:** Clinical sign observed in field studies in animals with different disorders linked to PCV3.

**Clinical signs**	**Tested samples**	**Country**	**References**
**Respiratory disorders**			
Mild and severe respiratory diseases.	Pulmonary homogenate/oral	USA	Phan et al. (10)
Moderate and diffuse lymph histiocytic interstitial dyspnea/pneumonia	Lung tissues	China	Palinski et al. (11)
Acute bronchitis	Tissues/Serums	China	Qi et al. (15)
**Gastrointestinal disorders**			
Diarrhea	Fecal specimens	China	Zhai et al. (54)
Diarrhea/vomiting	Intestinal tissues and lung tissues	China	Qi et al. (10)
Digestive disorders/catarrhal enteritis and catarrhal colitis	serum	Spain	Saporiti, et al. (69)
**Reproductive disorders**			
Reproductive failure	Serum/ Semen	China	Zou et al. (57)
Mortality of sows (aborted mummified fetuses)	Tissue pool from aborted fetus and stillborn piglet	Brazil/ Italy	Dal Santo et al. (67)Faccini et al. (64)
Sows giving birth to stillborn piglets	Tissues from mummified fetuses	USA	Arruda et al. (70)
**Neurological disorders**			
Congenital tremors	Brain tissue	China	Chen et al. (19)
Congenital tremors, neurological signs in piglets after birth	Brain tissue	United Kingdom	Williamson et al. (71)
Tremors, newborn piglets born weakly/myocarditis, encephalitis, gliosis, and lymphocytic perivascular cuff	Brain tissue	USA	Arruda et al. (70)
**Other disorders**			
Myocarditis/periarteritis	Different tissues	USA	Phan et al. (10)
Porcine Dermatitis and Nephropathy Syndrome	Tissues from kidneys and spleen	USA	Palinski et al. (11)
Acute deaths/myocarditis	Kidneys and spleen	Russia	Yuzhakov et al. (72)
Arteritis/systemic inflammation	Kidneys and spleen	Russia	Yuzhakov et al. (72)

PCV3 has been linked to reproductive issues and has a high potential for vertical transmission. A study by Zheng et al. (29) analyzed 222 tissue samples from stillborn fetuses in China and found that most samples tested negative for PCV2, but viral metagenomic sequencing revealed the presence of PCV3 in 59.5% (132/222) samples. Another study aimed to investigate PCV3’s association with reproductive issues in healthy sows. They analyzed serum samples from 85 sows with a history of reproductive disorders and 105 healthy sows, with a significantly higher PCV3 positive rate found in sows with reproductive failure (45.9%) than in healthy ones (21.9%) (57). In Brazil, a study conducted by Dal Santo et al. (67) found that 270 out of 276 mummified fetuses tested positive for PCV3. Similarly, in Argentina, mummified and stillborn fetuses resulting from reproductive failures showed 100% positivity for PCV3 (44).

Retrospective studies have revealed that PCV3 has been circulating in Poland since 2014, in Ireland since 2002, in Spain and China since the 1990s (20, 39), and in Brazil since 1967 (40). These findings suggest that the virus has been present in pig populations for decades prior to its initial reporting, regardless of whether it is linked to clinical symptoms.

Some studies have shown the still unknown impact of PCV3 on public health (14, 20). PCV1 and PCV2 DNA have already been found in vaccines intended for human use, probably originating from reagents of porcine origin in their manufacture, in addition to detection in samples from children who received live vaccine against rotavirus (76). It is known that PCV2 can infect human cells, both normal and cancerous, therefore, as it is a virus of the same genus, it may be that PCV3 can infect humans, a fact that still needs to be further studied. Recently, a herd of triple genetically modified pigs generated for xenotransplantation observed a sudden introduction of PCV3. These pigs served as donors for orthotopic heart transplants in baboons, with four cases involving PCV3-positive hearts resulting in transmission of the virus to the recipients. PCV3 was detected in all organs of the baboons, and a higher viral load was found in animals with a longer survival time, indicating replication of the virus. This marks the first reported instance of PCV3 trans-species transmission to baboons through heart transplantation from a PCV3-positive pig donor (68).

In a study conducted in a specific region of China, Porcine Circovirus 3 (PCV3), an emerging virus associated with swine dermatitis and nephropathy syndrome in swine, was thoroughly investigated for its prevalence and genotypic distribution. A total of 1,291 samples were collected from 211 pig farms in 15 provinces and municipalities in the region in question. Of these samples, 312 were positive for PCV3 using PCR, and a subsample consisting of 164 of these positive samples was subjected to sequencing and analysis (34). The results revealed that the overwhelming majority (61.8%) of the sequenced isolates belonged to the PCV3c genotype. Notably, the PCV3c genotype also emerged as predominant in Hubei, Hunan, Hebei provinces and Chongqing city. Furthermore, the analysis identified three sites under positive selection, located within the predicted epitope peptide, suggesting that porcine immunity may be influencing this highly positive selection. These findings are of significant relevance for understanding the spread of PCV3 in the studied region, as well as for continued research and development of control strategies (34).

A study carried out in the provinces of Sichuan and Gansu, China, with the purpose of evaluating the frequency of detection of Porcine Circovirus 3 (PCV3) in Tibetan pigs in three different provinces surrounding the Qinghai-Tibet plateau, it was observed that the prevalence of virus was significantly higher in samples from pigs with diarrhea compared to samples from healthy animals. Phylogenetic analysis of Cap proteins revealed the presence of three distinct clades among the 20 PCV3 strains, comprising PCV3a (40.00%), PCV3b (25%), and PCV3c (35.00%). These results highlight the prevalence of PCV3 in Tibetan pigs in high-altitude regions in China, highlighting the higher prevalence rates of PCV3a and PCV3b subtypes in samples from pigs with diarrhea. Such observations emphasize the need to consider PCV3 genotypes in research related to its pathogenicity, highlighting the importance of surveillance and control of this virus at a regional and global level (15).

Another study carried out in Vietnam investigated the genetic diversity of PCV3 in 249 pig samples from eight provinces. About 11.65% of the samples contained PCV3. Genetic analyzes revealed that 23 samples were of the PCV3b subtype and six belonged to subtypes c and a (a-1 and a-2). The sequences were highly similar (96.90-100% in genome and 96.19-100% in amino acids), and fifteen amino acid substitutions were found in the capsid proteins of Vietnamese PCV3 strains, contributing to the understanding of regional genetic diversity (77).

Some studies have shown curiosity about the still unknown impact of PCV3 on public health (13, 15, 34) PCV1 and PCV2 DNA have already been found in vaccines intended for human use, probably originating from reagents of porcine origin in their manufacture, in addition to detection in samples from children who received live vaccine against rotavirus (DOI: 10.4161/hv.26731) (80). It is known that PCV2 can infect human cells, both normal and cancerous, therefore, as it is a virus of the same genus, it may be that PCV3 can infect humans, a fact that still needs to be further studied.

## Clinical signs and pathology

PCV3 has been described and continues to be detected in pigs that exhibit a range of clinical signs including respiratory and digestive disorders, neurological alterations, cardiac and multisystemic inflammation, and reproductive disorders, as well as conditions like PDNS (10, 11, 46).

However, the mere presence of PCV3 genome in a diseased animal does not necessarily imply that the virus is the causative agent of the clinical signs or lesions observed. Most of the studies carried out so far lack a proper negative control to compare viral infection and disease (46). Furthermore, the detection of PCV3 through PCR in tissue, blood, and serum samples is frequently reported, but without any correlation with the animal’s lesions. In other words, few studies have yet demonstrated the virus’s presence in the lesions (20, 46, 78).

Arruda et al. (70) conducted a study on pigs from various farms in the United States at different production stages to detect the presence of PCV3 in lesions using histopathology and *in situ* hybridization (ISH) techniques. The results of the study suggested that PCV3 could be a potential cause of multisystemic inflammation and reproductive failure in pigs during the perinatal, growth, and termination phases. Several other studies have also detected PCV3 in lesions using ISH (10, 36, 46, 70–86).

There have been limited experimental investigations conducted on PCV3. In a study by Jiang et al. (74), 4 and 8-week-old SPF piglets were inoculated with PCV3. In the context of this experimental study involving PCV3 infection, the inoculum was obtained from clinical samples from pigs diagnosed with the infection. Tissues such as lungs, spleen and lymph nodes were collected post-mortem from infected pigs. The genetic material containing PCV3 was then extracted from these tissues using homogenization and processing techniques, followed by a purification step. The purified genetic material was amplified in cell culture or by the PCR technique to obtain an adequate quantity of the virus. This resulting inoculum, containing PCV3, was used for experimental infection in pigs, allowing the investigation of clinical, pathogenic and immunological aspects associated with PCV3 infection. All piglets that received PCV3 at 4 weeks old, exhibited symptoms such as fever, anorexia, coughing, sneezing, diarrhea, lethargy, rubefaction on the skin and ears, multifocal papules, shivering, and/or hyperspasmia. Among this group, two piglets displayed severe clinical signs, cardiac pathologies, multisystemic inflammation, and died during the experiment. In 8-week-old piglets administered with PCV3, typical clinical signs of PDNS were observed, although all the piglets survived for the entire duration of the study (28 days). Immunohistochemical staining detected PCV3-positive cells in various tissues and organs (including the lung, heart, kidney, lymph nodes, spleen, liver, and small intestines) of PCV3-inoculated piglets. The peak of viremia (approximately 7.72 × 10^8^ PCV3 copies/mL) in the 4-week-old PCV3-inoculated piglets was detected at 21 days post-inoculation (dpi). Similarly, the highest level of PCV3 (7.92 × 10^7^ copies/mL) in the 8-week-old PCV3-inoculated piglets was also detected at 21 dpi. Mora-Diaz et al. (87) inoculated colostrum-deprived (CD/CD) pigs that were 6 weeks old. PCV3 was isolated from multiple tissues, including the lung, kidney, heart, and brain of perinatal pigs with encephalitis and/or myocarditis, and stillborn and mummified fetuses. Although all animals that were infected with the PCV3 isolate remained clinically healthy throughout the study, histological evaluation revealed lesions of ongoing multisystemic inflammation, lymphoplasmacytic myocarditis, and periarteritis in 4 out of 8 pigs. PCV3 replication was confirmed by *in situ* hybridization (ISH). The study also demonstrated lymphoplasmacytic interstitial nephritis and periarteritis in kidney tissue, and lymphoplasmacytic periarteritis and arteritis of the intestinal serosa of PCV3-inoculated pigs. PCV3 replication was also confirmed within inflammatory cells, the tubular renal epithelium, endothelial cells, and the tunica media of arteries. Viremia was first detected at 7 days post-inoculation (dpi) and was detected in all animals by 28 dpi.

Continuing to explore alternative animal models beyond pigs, (60)realized a study employing infectious clones of PCV3 to induce infection in Kunming mice. They successfully demonstrated the pathogenic potential of the acquired infectious clones of PCV3, establishing Kunming mice as an animal model. Findings from RT-PCR, Western blotting, and ISH highlighted the ability of PCV3 to infect both the myocardium and alveoli of Kunming mice.

The alterations within the pulmonary and cardiac tissues revealed a proliferation of alveolar epithelial cells in the local lung region, resulting in congestion at the periphery of the lobules. Notably, strong positive reactions for PCV3 were evident in the lung. Within the heart, intense positive reactions for PCV3 were detected in necrotic tissues and vascular content, (74) emphasized the significance of their findings by detecting PCV3 in laboratory mice. This underscores the necessity for testing all animal species used in experimental infections, highlighting the importance of thorough screening protocols.

## Coinfections

Due to its global spread, there have been reports of co-infections involving PCV3. Given that PCV2 and PCV3 are from the same genus and considering that co-infections of PCV2 with other pathogens have been shown to worsen the disease both in field and experimental settings, it is possible that PCV3 infection may also facilitate co-infections with other disease-causing agents in pigs (14, 20, 46, 19).

Previous studies have shown that *Porcine circovirus* 3 (PCV3) can co-infect with various other pathogens such as Porcine Reproductive and Respiratory Syndrome Virus (PRRS), Porcine Epidemic Diarrhea Virus (PEDV), *Torque teno sus virus* (TTSuV; comprise TTSuV1a and TTSuVκ2a), *Porcine parvovirus* (PPV), Virus of Classical Swine Fever (CSFV), Atypical porcine pestivirus (APPV), Porcine pseudorabies virus (PRV), *Streptococcus* spp., *Mycoplasma hyopneumoniae*, and *Leptospira* spp. The co-infection prevalence rates of these pathogens with PCV3 range from 5% (PRV) to 100% (*Mycoplasma hyopneumoniae*) (15, 26, 29, 30, 34, 45, 36, 53, 61, 66, 81, 86, 88–90).

**Figure 4:**
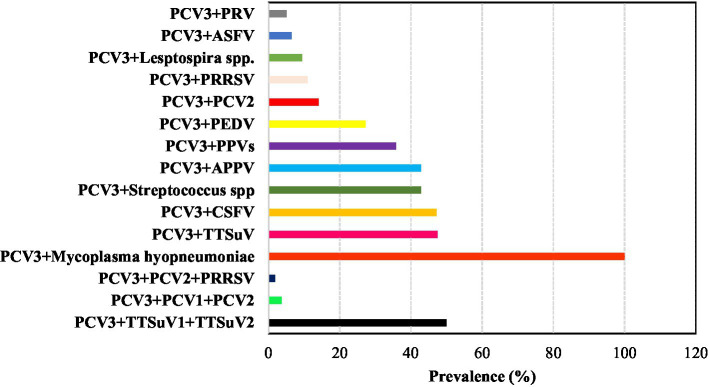
Overall prevalence (%) of pathogens involved in co-infections with PCV3 based on consulted literature.

Other studies have reported triple co-infections involving PCV3, PCV2, and PRRSV; PCV3, PCV1, and PCV2; and PCV3, TTSuV1, and TTSuV2, with prevalence rates of 1.9, 3.6, and 50%, respectively (31, 56, 91). Moreover, the recent discovery of PCV3 in Mozambique, Africa revealed co-infection with the African Swine Fever Virus (ASFV), where 6 out of 7 positive PCV3 samples also tested positive for ASFV (6.5% of total samples) (58) (Figure 4).

Although co-infections are not well understood, PCV3 is widely prevalent in healthy animals and previous experimental studies have shown that the virus can cause lesions and clinical symptoms, with the age of infection being a significant factor. However, the impact of co-infections remains uncertain. Nevertheless, as observed with PCV2, co-infections may lead to severe clinical symptoms observed in field studies.

## Diagnosis

The most used detection methods so far are: *in situ* hybridization (ISH), quantitative polymerase chain reaction (PCRq) and enzyme-linked immunosorbent assay (ELISA) (29, 46, 92–95) (Table 3).

**Table 3:** Laboratory methods for PCV3 detection in field studies from various samples in different countries.

**Method**	**Country**	**Sample**	**No. of samples**	**Positive samples**	**References**
**PCR**	Poland	Serum	1,050	268 (25.5%)	Stadejek et al. (50)
	Northern Ireland	Tissue and feces	240	48 (20%)	Collins et al. (48)
	England	Tissue	80	4 (5%)	Collins et al. (48)
	Italy*	Tissue/tissue serum and nasal swabs	16/99	2 (12.5%)/37 (37.4%)	Faccini et al. (64)Franzo et al. (47)
	Denmark	Tissue and serum	78	44 (56.4%)	Franzo et al. (47)
	Spain**	Serum/Serum/Tissue	94/ 654/300	14 (14.9%)/75 (11.5%)/74 (24.6%)	Franzo et al. (47)/Klaumann et al. (97) /Ruiz et al. (78)
	Sweden	Tissue	49	10 (20.4%)	Ye et al. (27)
	United Kingdon	Serum	126	8 (6.3%)	Feng et al. (50)
	France	Serum	139	9 (6.5%)	Feng et al. (50)
	Hungary*	Tissue/Serum and oral fluid	330/2.128	129 (43%)/558 (30.8%)/	Deim et al. (42)/Igriczi et al. (79)
	Japan	Tissue	73	7 (9.6%)	Hayashi et al. (80)
	Korea	Tissue	690	57 (9.8%)	Kim et al. (81)
	China**	Tissue/Tissue/Tissue and blood	105/616/535	35 (33.3%)/75 (12.2%)/216 (40.4%)	Ha et al. (66)/Qi et al. (15)/Zou et al. (23)
	Thailand	Serum and tissue	79	29 (36.7%)	Sukmak et al. (82)
	United States**	Serum/Tissue and serum	36/336/2,177	22 (61.1%)/37 (11.1%)/577 (27%)	Arruda et al. (70)/Feng et al. (47)/Yang et al. (83)
	Brazil**	Tissue/tissue and FFPE/Tissue	67/35/ 143/276	32 (47.8%)/ 14 (40%); 12 (8.4%)/270 (97%)	Saraiva et al.(35) /Rodrigues et al. (39)Dal Santo et al. (84)
	Colombia	Serum and tissue	8	2 (25%)	Vargas-Bermúdez et al. (36)
	Chile	Tissue	50	35 (70%)	Rubilar et al. (43)
	Argentina	Tissue	203	18 (8.9%)	Serena et al. (41)
	Mozambique	Tissue	91	7 (7.5%)	Anahory et al. (55)
**ISH**	United States	Serum/Tissue and serum	5	5 (100%)	Arruda et al. (70)
	Brazil	Tissue	11	6 (54.5%)	Molossi1 et al. (85)
**ELISA**	China*	Serum	190	64 (33.8%)	Zhang et al. (67)
	China	Serum	2568	1101 (42.7%)	Ge et al. (16)
	China	Serum	1,688	840 (49.8%)	Deng et al. (92)

*= data comprise two studies; **= data comprise three studies; FFPE = formalin-fixed paraffin-embedded; /= used to separate information from different studies.

PCRq analysis has been widely used to analyze the tissues collected from field cases. In Hungary, viral DNA was detected in fetal and neonatal thymus (89%; 49/55), lymph nodes (60%; 33/55), placenta (50.9%, 28/55), spleen (21.8%, 12/55), kidneys (7.2%, 4/55), and liver (5.4%, 3/55), indicating a high prevalence of PCV3 in reproductive failure cases (42). The highest viral loads were reported in fetal or neonatal heart tissues, ranging from 10^9^ to 10^12^ genomic copies/gram, followed by fetal kidneys and lungs, with loads ranging from 10^9^ to 10^11^ genomic copies/gram (91, 96). High viral loads of PCV3 have also been observed in brain tissues of cases with congenital tremors (96). Viral DNA of PCV3 was detected in semen, serum, feces, colostrum, and oral fluid, with viral loads ranging from 10^2.5^ to 10^7.2^ copies/mL (12, 26, 97–99).

Different laboratory techniques are available for the detection of PCV3 antigen and/or nucleic acid. For instance, *in situ* hybridization (ISH) has been used to identify the virus in the intestine, heart, and lung (26, 70, 81), while immunohistochemistry (IHC) has been used to detect the virus in the lungs, lymph nodes, kidneys, liver, and heart (74).

Unfortunately, information about PCV3 remains limited and few studies have successfully elucidated the cause of the disease associated with this new virus. This shortage of data may hinder diagnosis, as the overall picture of the infection has not been fully established (20, 46).

To make an accurate diagnosis, it is crucial to analyze clinical signs or evidence of decreased productivity, the evolution of events that may interfere with the onset of the problem, and the detection of the pathogen associated with the lesions present in the animals at the onset of the disease. This latter factor is particularly important for certain infectious agents, such as PCVs.

Detection of the PCV3 virus in pigs involves the collection of several samples, the most common being blood, serum, tissues such as liver and lymph nodes, as well as saliva and feces samples. To research this virus, laboratory techniques such as polymerase chain reaction (PCR), real-time PCR (qPCR), real-time reverse transcriptase PCR (RT-qPCR) and *in situ* hybridization techniques are widely used. Furthermore, histopathological and immunohistochemical analysis of tissues also play a fundamental role in the identification and characterization of PCV3 in pigs, contributing to the understanding of the epidemiology and pathogenesis of this viral infection in pig farms.

## Conclusion

As final considerations, PCV3’s extensive genetic diversity is evident through the multitude of genotypes identified thus far. It has been documented across nearly all continents, except for Oceania and Antarctica. Its detection in various animal species shows its one health implications and the potential for zoonotic transmission. Worthy of significant consideration. While linked with diverse clinical manifestations, PCV3’s most notable impact remains on reproductive disorders. Recognizing the significance of asymptomatic carriers-whether swine or non-swine-is crucial, as they serve as potential reservoirs for the virus. Field studies shows co-infections with other microorganisms, yet the role of these co-agents in initiating or exacerbating clinical signs, whether mild or severe, lacks conclusive association. Various sample types can be utilized for PCV3 detection, with molecular tests like PCR and qPCR being the most used to detect the virus. However, non-molecular tests play an important role in establishing the association between the agent and lesions alongside tests demonstrating viral replication (such as RT-qPCR and RNA-ISH). Finally, since its discovery, PCV3 has proven to be a significant and challenging emerging agent in global swine production. Like PCV2, it displays a high capacity for mutation, potentially exacerbating clinical signs when combined with other agent and due its extensive distribution.

## Author contributions

DS: Methodology, Writing – review & editing. RS: Data curation, Investigation, Writing – original draft, Writing – review & editing. VS: Data curation, Investigation, Writing – review & editing. AC: Funding acquisition, Project administration, Software, Supervision, Validation, Writing – original draft, Writing – review & editing.

## Footnotes

^1^https://pubmed.ncbi.nlm.nih.gov/
