# Corrigendum: Porcine circovirus 3: a new challenge to explore

**DOI:** 10.3389/fvets.2024.1448165

**Published:** 2024-07-29

**Authors:** Rosecleer Rodrigues da Silva, Diego Ferreira da Silva, Victor Hugo da Silva, Alessandra M. M. G. de Castro

**Affiliations:** ^1^Department of Undergraduate Studies in Veterinary Medicine, Faculdade Anclivepa, São Paulo, Brazil; ^2^Postgraduate Program in Adult Health Nursing (PROESA), Escola de Enfermagem da Universidade de São Paulo (EEUSP), São Paulo, Brazil; ^3^Graduate Program in Environmental and Experimental Pathology, Universidade Paulista (UNIP), São Paulo, Brazil

**Keywords:** emerging, porcine circovirus, swine, PCR, hosts

In the published article, there was an error.

A correction has been made to **Characterization and viral diversity**, Paragraph 3. This sentence previously stated:

“Therefore, future research utilizing advanced techniques is essential to elucidate the complexities of ORF1 in PCV4 and its implications for swine health”

This sentence is going to be removed as this is not relevant to the context.

A correction has been made to **Coinfections**, Paragraphs 5 and 6. These paragraphs stated:

“Continuing to explore alternative animal models beyond pigs, (60) realized a study employing infectious clones of PCV3 to induce infection in Kunming mice. They successfully demonstrated the pathogenic potential of the acquired infectious clones of PCV3, establishing Kunming mice as an animal model. Findings from RT-PCR, Western blotting, and ISH highlighted the ability of PCV3 to infect both the myocardium and alveoli of Kunming mice. The alterations within the pulmonary and cardiac tissues revealed a proliferation of alveolar epithelial cells in the local lung region, resulting in congestion at the periphery of the lobules. Notably, strong positive reactions for PCV3 were evident in the lung. Within the heart, intense positive reactions for PCV3 were detected in necrotic tissues and vascular content, (74) emphasized the significance of their findings by detecting PCV3 in laboratory mice. This underscores the necessity for testing all animal species used in experimental infections, highlighting the importance of thorough screening protocols.”

These paragraphs were moved to **Clinical signs and pathology** after paragraph 4.

A correction has been made to **Conclusion**, Paragraph 1. This sentence previously stated:

“Na linha 1043 excluir o parágrafo e acrescentar este:”

This sentence has been removed.

The authors apologize for these errors and state that these does not change the scientific conclusions of the article in any way. The original article has been updated.

